# Good nights sleep program: design and preliminary findings from a randomized clinical trial to improve child and parent sleep in low-income families

**DOI:** 10.3389/frsle.2026.1764378

**Published:** 2026-06-17

**Authors:** Brian T. Gillis, Ben Hinnant, Olivia Martín-Piñón, Elise L. Neuhoff

**Affiliations:** 1 Department of Human Development and Family Science, Auburn University, Auburn, AL, United States; 2 Department of Psychology, Arizona State University, Tempe, AZ, United States

**Keywords:** childhood, intervention, low income, parent, sleep, sleep environment, sleep hygiene, sleep hygiene and environment

## Abstract

**Introduction:**

Sleep is essential for human health. For low-income individuals and families, sufficient, high-quality, regular sleep can be difficult to obtain due to two main barriers: factors that prevent optimum sleep hygiene behaviors and aspects of the bedroom environment, such as noise and temperature, that reduce sleep. This article describes the rationale and design of the Good Nights Sleep Program, a pilot of a randomized clinical trial (clinicaltrials.gov/study/NCT06249217) that combines education with behavior change strategies that lead children and their parents to select, implement, and track changes to their sleep behaviors and environments. Following the conceptual description of the interventions, descriptive statistics from the pilot study are presented.

**Methods:**

The study enrolled parent-child dyads with a mean family income-to-needs ratio of 1.68 (75% Black; 25% White).

**Results:**

Findings provided proof of concept for the intervention and descriptive preliminary evidence. Children receiving the intervention had longer actigraphy-derived sleep hours compared to waitlist control-arm participants, and parents had shorter self-reported usual sleep latency and more consistent actigraphic wake times.

**Discussion:**

The Good Nights Sleep Program offers a promising model for empowering children and parents to make attainable changes that yield benefits for their sleep.

## Introduction

Sleep is as necessary to life as food, water, and shelter (Maslow, 1943), and improving sleep is an essential objective of Healthy People 2030 (U.S. Department of Health and Human Services, 2020) and the National Institutes of Health Sleep Research Plan (National Heart, Lung, and Blood Institute, 2021). Sleep problems are highly prevalent in childhood and adulthood (Grandner, 2022) and can influence cognitive, physical, and mental health, as well as children's psychosocial and academic development (Short et al., 2018; Matricciani et al., 2019). Notably, children from economically disadvantaged families suffer disproportionately from inadequate sleep: they have shorter and poorer objective, actigraphy-derived sleep and greater subjective sleep problems compared to children in wealthier families (Etindele Sosso et al., 2021; Fuligni et al., 2021). The link between low family income and poorer sleep health extends to adulthood as well (Grandner et al., 2016).

A consistent body of research has demonstrated that low-income children and parents face irregularity in family schedules, high family stress, and physical/built environments that disrupt sleep, such as non-ideal sleeping temperatures, nighttime light, and loud noise (Jones and Bucchio, 2018; Philbrook et al., 2020; Crea, 2021; Hinnant et al., 2023). This creates significant barriers to behaviors that support sleep regularity and impacts environmental factors that reduce the length and quality of sleep (Grandner et al., 2016; Covington et al., 2021; Etindele Sosso et al., 2021; Fuligni et al., 2021).

**Figure 1:**
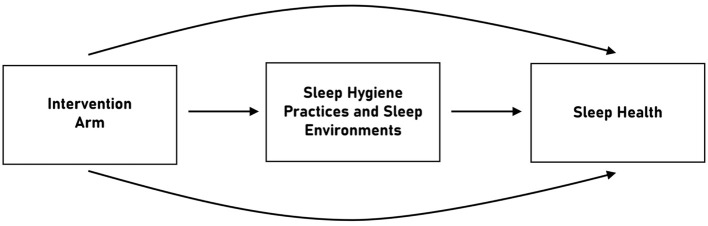
Conceptual model.

Basic research in this area converges on two potential targets of intervention to improve sleep health for individuals within low-income families: sleep hygiene practices and sleep environments. However, many sleep interventions are prescriptive, addressing sleep problems that are applied in the same way to all individuals. Adaptive interventions that identify and target specific problems with sleep hygiene practices and sleep environments may be more effective (Fischer et al., 2021; Adams et al., 2023). We created and pilot-tested the Good Nights Sleep Program, a novel intervention to meet these needs. The intervention adapts established, evidence-based health behavior change strategies to collaboratively identify and address individual- and dyad-level sleep health behavior and sleep environment problems among low-income families (Figure 1). In this paper, we lay out the need for such adaptive interventions, explain the theoretical rationale behind our intervention, describe the study design, and report exploratory, preliminary findings.

Sleep health is linked to broader health. Poor sleep health (i.e., short, fragmented, or inconsistent sleep) is linked to physical illness (Patel et al., 2010; Grandner et al., 2016; Billings et al., 2021) and impairments in psychological and cognitive functioning (Moore et al., 2002; Short et al., 2018; Williams et al., 2019; El-Sheikh et al., 2022). These links contribute to the development of chronic health conditions such as hypertension and diabetes (Jean-Louis et al., 2022). Suboptimal sleep hygiene practices and sleep environments are pathways through which poverty is associated with physical illness (Bagley et al., 2015; McWood et al., 2022; Hinnant et al., 2023), further underscoring the need for efficacious and effective interventions that improve sleep health among low-income families while acknowledging the reality of their barriers, such as limited access to the material and monetary resources needed for optimal sleep hygiene.

The sleep of parents and children is intertwined. Challenges in behavioral and environmental domains are experienced not only at an individual level (i.e., children and parents individually) but also at a dyadic level (i.e., children and parents together). Behaviorally, greater screen time and less physical activity among parents is associated with shorter sleep among children (Rea et al., 2016), while child sleep problems—even in grade-school—are associated with disruptions in parents' nighttime sleep and daytime sleepiness (Boergers et al., 2007). Environmentally, detrimental aspects of sleep environments such as noise and temperature can affect whole households, even if children and parents have separate bedrooms (Hoyniak et al., 2022). Acknowledging and addressing the interdependent nature of sleep hygiene, sleep environments, and sleep health of individuals within families is an important part of family-focused sleep interventions (Johnson et al., 2018; Jackson et al., 2020).

### Theoretical rationale

For the past 75 years, the Health Belief Model (HBM) has been consistently cited in health behavior research, and it remains a foundational framework for understanding why people engage in health-promoting behaviors (Alyafei and Easton-Carr, 2024). Our novel sleep health intervention integrates four primary cognitive constructs within this framework: (1) perceived benefits, (2) perceived barriers, (3) cues to action, and (4) self-efficacy. Our innovative approach to enhancing parent-child dyad-level sleep health adapts these evidence-based dimensions of health behavior change by educating children and parents on the benefits of quality sleep, identifying possible barriers and personalized solutions to goal pursuit, completing daily diaries, and self-monitoring progress toward specific, measurable, achievable, relevant, and time-bound (SMART) goals (Bjerke and Renger, 2017) to promote positive sleep health behavior modifications in both children and parents.

A multitude of obstacles may jeopardize an individual's commitment to a recommended health behavior: pain or physical discomfort, social consequences, cost, and accessibility are among the most commonly perceived downsides to health behavior change (Glanz et al., 2008; Carpenter, 2010; Alyafei and Easton-Carr, 2024). Accordingly, our intervention includes anticipated barrier identification coupled with empathy and personalized solutions to overcome barriers to leverage the predictive value that such obstacles have to sustained health behavior change (Glanz et al., 2008).

When the mind discerns perceived barriers, it simultaneously weighs them against expected benefits in an often-unconscious cost-benefit analysis (Glanz et al., 2008). Therefore, perceived benefits cannot be overlooked (Carpenter, 2010). Perceived benefits refer to the expected risk reduction against the target health threat, as well as the anticipated non-health-related advantages (e.g. relational, financial) (Glanz et al., 2008; Carpenter, 2010; Alyafei and Easton-Carr, 2024). With this in mind, the educational component of our intervention is focused on benefits of good sleep, highlighting for children and parents what they hope to gain from making changes to sleep hygiene practices and sleep environments.

Our intervention's focus on low-income families meant that economic constraints and distress can be a significant barrier to healthy sleep and lasting behavior change for some families. Ideal sleep hygiene is out of reach for many families in the United States, especially for low-income families (Gregory et al., 2025). The high cost and inaccessibility of stable and safe housing, heating and cooling homes, and realities of non-standard work hours limit families' ability to engage in routines and create an ideal sleep environment (Allen et al., 2016; Craft et al., 2021; Crea, 2021). Psychological stress of struggling to make ends meet can increase pre sleep arousal and can spillover to impact child sleep health (Bagley et al., 2015). This intervention seeks to address economic barriers head-on and with intention.

Our intervention is further strengthened through its alignment with two constructs later added to the HBM, cues to action and self-efficacy (Alyafei and Easton-Carr, 2024). Cues to action involve the addition of stimuli, either internal or external, to initiate a preventive health behavior (Carpenter, 2010; Alyafei and Easton-Carr, 2024). Notably, an individual's experience with a cue can interrupt their present perceptions, stimulate a broader evaluation of that individual's present life circumstances, and trigger the recall of previous health education material (Meillier et al., 1997). The Good Nights Sleep Program's educational component is bolstered by this HBM domain as families take home educational materials with them in hand and additionally complete a daily log of sleep goals for both the child and the parent, providing in-home cues to action.

The Good Nights Sleep Program rests on the belief that families can make their own decisions around goals related to sleep hygiene practices and sleep environments. The program empowers children and parents to choose what they want to work on and supports them in making such changes themselves, thus reinforcing self-efficacy, an individual's belief in their capacity to adhere to a preventative health behavior (Alyafei and Easton-Carr, 2024).

The inclusion of parent-child dyads optimizes the family-level integration of health behavior change through observational learning, a foundational dimension of the Social Cognitive Theory (SCT), which posits that behavior is an interaction between people and their surroundings (Islam et al., 2023). Specifically, children may have enhanced desire to engage in sleep health behaviors when these behaviors are modeled by parents (Islam et al., 2023). Alongside observational learning, SCT constructs include behavioral capability, reinforcement, and self-control. However, the most utilized SCT construct, also emphasized in the HBM, is self-efficacy (Islam et al., 2023; Alyafei and Easton-Carr, 2024). This shared domain across the HBM and SCT reinforces the present intervention's efforts to increase families' beliefs in their capability to change sleep environment and behavior through personalized solutions, sleep health education, and sleep hygiene items (Islam et al., 2023; Alyafei and Easton-Carr, 2024).

Taken together, the constructs of the SCT support our intervention's hallmark systemic change process in which behavior is observed, learned, and adopted through interaction with other individuals (Islam et al., 2023; Hasan et al., 2024). Children adjust behavior to align with a parent (i.e., observational learning) and demonstrate an inclination to repeat that behavior change when they witness the positive results of that change in the participating parent (i.e., reinforcement learning) (Islam et al., 2023; Hasan et al., 2024). Our intervention's design for parent-child dyads to experience sleep hygiene changes in tandem is a strength of this process. Last, in addition to HBM and SCT, our intervention is enhanced with cognitive-behavioral components (e.g., emphases on sleep hygiene practices and sleep environments) (Blake et al., 2017) and models of sleep health disparities and resilience (e.g., needs to improve accessibility, provide sleep-support resources, and ensure cultural appropriateness) (Mindell et al., 2016; Billings et al., 2021; Adams et al., 2023).

### Program design

The design of the program takes into account barriers to intervention utilization and seeks to overcome them by being (1) easily accessible, (2) sensitive to context, and (3) tailored to specific sleep impediments (Fischer et al., 2021; Adams et al., 2023). Our intervention adapts evidence-based sleep improvement strategies to the needs of low-income families by following each of these guidelines. First, the intervention is easily accessible by being scheduled at times selected by families in afternoons, evenings, and weekends, taking just 5 h to complete over the course of a month. When needed, research assistants provide childcare for non-participant family members such as siblings. Second, the intervention is sensitive to context. Qualitative interviews with low-income adults have suggested that adaptation of sleep interventions to low-income families should support parents in helping children with sleep behaviors by pre-planning action and must address physical challenges in the sleep environment such as light, temperature, and noise (Rottapel et al., 2020; Adams et al., 2023). A review of health promotion interventions for low-income groups further identified the need to focus on a small number of changes and to combine education with goal setting and barrier identification (Michie et al., 2009). Our intervention asks each participant to select just two things they want to do differently for their sleep hygiene and environment rather than providing a long list of “shoulds” to improve sleep. We provide brief sleep health education and then facilitate goal selection with a discussion of anticipated barriers to help parents and children plan ways to overcome them. These specific techniques have been shown to work additively to increase motivation, to move from motivation to action, and to help low-income individuals attain realistic goals (Michie et al., 2009). Finally, the intervention is tailored to specific sleep impediments by asking participants to select sleep goals that are relevant for their needs.

## Materials and methods

### Participants

We recruited 15 parent-child dyads (30 total participants) who met inclusion criteria: (1) children were in third grade and eligible for or enrolled in a free or reduced-price lunch program at their school, used as a proxy for family economic disadvantage, and (2) could speak and read English or Spanish. Parent-child dyads were excluded if they met any of the following criteria: (1) parent or child diagnosed with a sleep disorder (e.g., sleep apnea, narcolepsy), (2) used medications that disrupt sleep (e.g., stimulants such as methylphenidate), or (3) used prescription medications for sleep (e.g., trazodone, zolpidem).

All parent participants were mothers^1^ (75% Black; 25% White) and were primary caregivers of their children. Parents' ages ranged from 28 to 51 years, with a mean of 40.10 (*SD* = 5.88) years. Children had a mode age of 9 years, with 69% assigned female and 31% assigned male at birth; child gender identity was not assessed (two male participants were age 8, nine female participants were age 9, and two male participants were age 10). Children lived primarily with their mother only (62%), followed by both parents (23%) or shared custody (15%). Most parents were employed (85%). Three-fourths (75%) of families lived in or near poverty, defined as less than two times the federal poverty line taking into account household size (U.S. Department of Commerce, 2025). Specifically, 33% of families had an income-to-needs ratio of < 1.00, 42% were between 1.00 and 2.00, 25% were greater than 2.00.

Of the 15 participant dyads, 13 completed the study, resulting in a retention rate of 87%. Busyness of family schedule was the reason of attrition for both families who did not complete the study. Families in the experimental arm reported meeting their SMART sleep goals on 82% of the nights between Weeks 2 and 4. All waitlist control families elected to receive the intervention at Week 4.

### Measures

#### Actigraphy

During the pilot study, children and parents wore Fitbit Inspire 3 devices (Fitbit Enterprise/Google, San Francisco, CA) from Week 0 to Week 4 while sleeping at home^2^. Data were collected in 30-s epochs, scored by Fitbit's proprietary algorithm (Bailey et al., 2025), processed by Fitabase (Small Steps Labs, San Diego, CA). Several established sleep parameters were assessed, representing various dimensions of sleep indexed using common definitions for actigraphy variables (Ancoli-Israel et al., 2015; Sadeh, 2015). **Sleep hours** (total sleep time exclusive of night wakings) represented sleep duration. **Sleep efficiency** (percent of time from sleep onset to wake that was determined to be sleep; elsewhere in the literature, this is referred to as sleep percent) and **wake after sleep onset** (WASO; number of minutes scored as wake between sleep onset and wake) was used to represent sleep quality. **Sleep onset** (clock time at start of main sleep period) and **wake** (clock time at end of main sleep period) was our indicator of sleep timing. Sleep variability was represented by **variability in sleep hours, onset**, and **wake** using the coefficient of variability, which, for each variable, is calculated by dividing each individual's standard deviation across nights by their individual mean across nights (Snedecor and Cochran, 1989). Sleep hours, WASO, onset, and wake were extracted by Fitabase; sleep efficiency was calculated by dividing sleep hours by the total time between onset and wake and multiplying by 100. A sleep diary (described below) guided actigraphy cleaning, in accordance with established guidelines (Ancoli-Israel et al., 2015); all actigraphy-derived sleep onset and wake times were within 30 min of self-reported times on sleep diaries. Data were included if participants had at least five nights of actigraphy at Weeks 0–2 and at least five nights of actigraphy at Weeks 2–4. Weeknight and weekend sleep data were used in analyses without disaggregation.

#### Semi-structured interview

Research assistants performed a semi-structured interview in Week 0 that covered families' activities on a typical weekday evening every hour starting from when the target child gets home from school to when the parent goes to bed. The interview also included questions about the sleep environment and waking up in the morning (see Table 1 for Session 1 interview schedule). Families in the experimental arm received a follow-up semi-structured interview in Week 4. The interview asked parents and children to assess their sleep goals and the intervention overall (see Table 2 for Session 3 interview schedule).

**Table 1:** Interview schedule for session 1 (week 0).

Content area	Question
Activities and routines	What time do you leave school? What time do you get home from school? What activities do you do in each hour from the time you get home to the time you go to bed? Please give me as much detail as possible about the hour before bed.
Sleep environment	What room in the house do you sleep in? Does anyone else sleep in that room? What time do you go to that room? Is there anything about your room or your house that makes it hard to *fall* asleep? Is there anything about your room or your house that makes it hard to *stay* asleep? Does anything get in the way of your sleep? If so, what?
Waking up	What time do you wake up in the morning? How easily do you wake up?

**Table 2:** Interview schedule for session 3 (week 4).

Content area	Question
Assessment of goals	Did the goals and associated strategies feel feasible in your daily life? *If not*, what got in the way? Or what would have made it easier? Did the goals feel relevant? Do you plan on continuing these sleep goals? Why or why not?
Assessment of intervention	How did it feel focusing on these things in your daily life? Is there anything that we did particularly well? Is there anything you found particularly helpful?

#### Sleep diary

Parents and children completed separate sleep paper sleep diaries between each lab session. Diaries asked families to report on their bed time, time to fall asleep (in minutes), and wake time for each night. This was used to corroborate sleep actigraphy. Families also reported medication use, if they were sick, if they took a nap that day, and if so, how long.

#### Sleep surveys

During each session (Weeks 0, 2, and 4) parents and children completed surveys of key sleep health dimensions on iPads. For children, subjective sleep quality was rated using the sleep/wake problems subscale of the School Sleep Habits Survey (Wolfson et al., 2003). Daytime sleepiness was measured using the Cleveland Adolescent Sleepiness Questionnaire (Spilsbury et al., 2007). Sleep hygiene behaviors were measured using Adolescent Sleep Hygiene Scale (Storfer-Isser et al., 2013). Sleep environment was measured using the Children's and Adolescents' Sleep Environment Scale (Peltz et al., 2022). Importance of sleep was measured using a single item, “On a scale from 1 to 10 (1 = not important at all, 10 = extremely important) how important do you think sleep is?” Sleep locus of control was adapted from the Health Locus of Control Scale (Wallston et al., 1976), the Multidimensional Health Locus of Control Scale (Wallston et al., 1978), and the Sleep Locus of Control Scale (Vincent et al., 2004).

Parents completed the Pittsburgh Sleep Quality Index (PSQI; Buysse et al., 1989) with questions adapted to assess the previous 2 weeks. Adult daytime sleepiness measured using the Epworth Sleepiness Scale (Johns, 1992). Sleep hygiene behaviors were measured using Adolescent Sleep Hygiene Scale (Storfer-Isser et al., 2013). Sleep environment was measured using the Children's and Adolescents' Sleep Environment Scale (Peltz et al., 2022). Parents reported on the extent to which they believed their actions could influence their child's sleep using an adaptation of the Health Locus of Control Scale (Wallston et al., 1976), Multidimensional Health Locus of Control Scale (Wallston et al., 1978), and Sleep Locus of Control Scale (Vincent et al., 2004).

### Procedure

**Figure 2:**
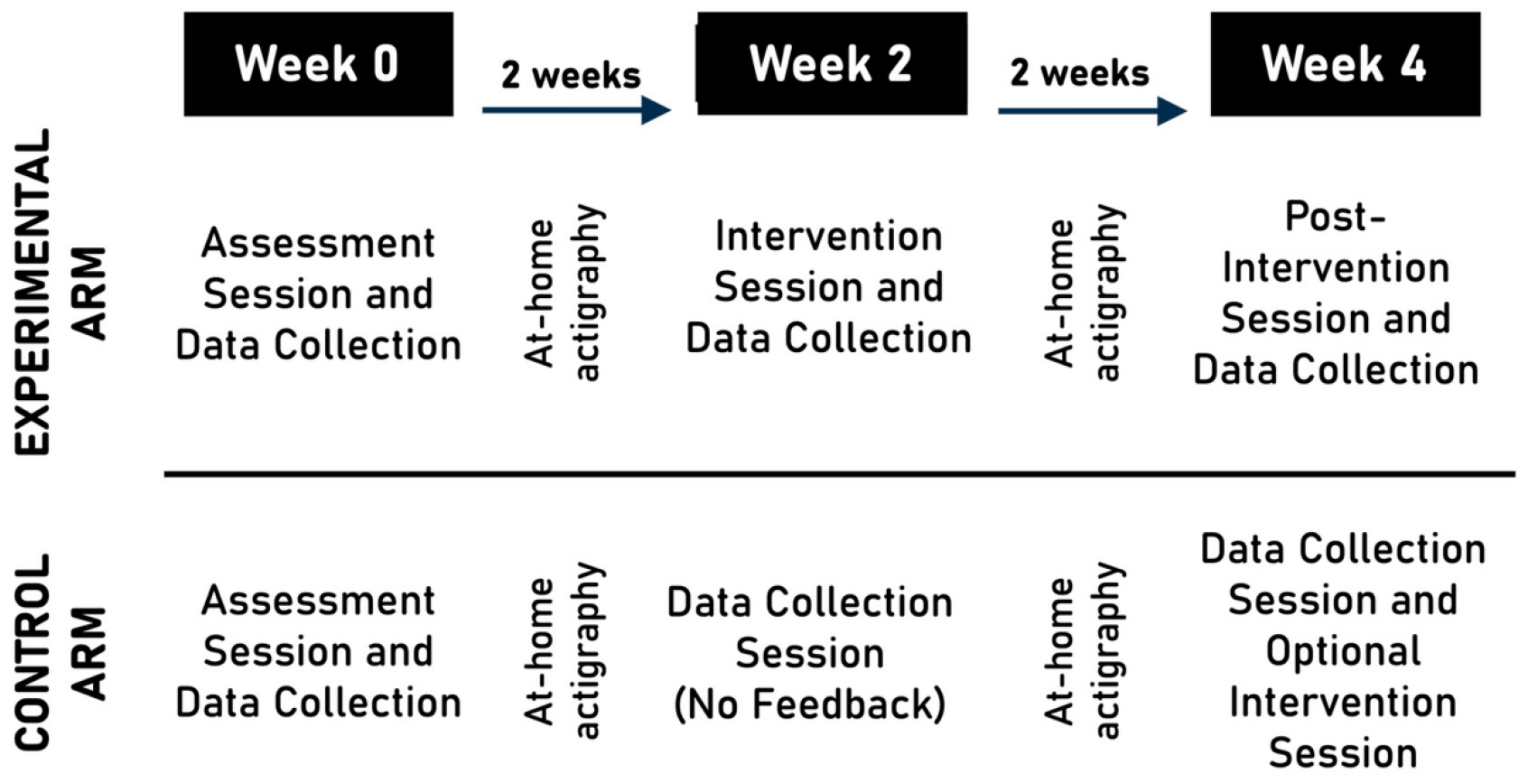
Treatment activities and timeline by arm.

Our pilot study, Stage 1 of the intervention, was registered with clinicaltrials.gov as NCT06249217 and approved by the Auburn University Institutional Review Board (protocol #23-562 MR 2311). The research design for the pilot study, depicted in Figure 2, was a two-arm (experimental, waitlist control) RCT with three repeated measures of key study variables over the course of 4 weeks. This was a methodologically rigorous design with high internal validity, allowing for causal inferences about any observed effects of the intervention. Random assignment to conditions helped to ensure that the experimental and waitlist control arms were equivalent prior to the intervention and that any subsequent changes are not attributable to initial group differences. The participant child and one participant parent dyad were randomly assigned to either the experimental or waitlist control arm.

In Week 0, we conducted a comprehensive baseline *assessment* of the sleep environments, sleep hygiene practices, subjective sleep health indicators, and cognitive, mental, and physical health. First, we complete a semi-structured interview that inquired about a family's schedule from the time the child gets home from school to lights out, perceptions of their sleep quantity and quality, and barriers to restorative sleep. As this is baseline data collection, no recommendations were provided at this step of the program. Next, parents and children completed surveys to report on more detailed aspects of the sleep environment such as sound, light, temperature, space, and bedding. Surveys also included measures for sleep hygiene practices such as sleep schedule, bedtime routine, screen use, physical activity, and eating and drinking around bedtime. Parents and children completed surveys independently, yet during the survey procedure were seated next to one another, and in certain instances, interacted with one another as needed (e.g., to ask questions). Finally, parents and children completed a brief cognitive test battery measuring attention, cognitive flexibility, and inhibitory control on a tablet. Finally, families were issued Fitbits to assess actigraphic sleep along with sleep diaries. Children and parents selected from a variety of colored wrist bands for their device to encourage individual expression and increase study engagement. Families were told they could wear the device all day, as long as they wore it while sleeping and the device remained charged. Families were given instructions on charging and syncing devices.

In between Weeks 0 and 2, RAs summarized families' interview and survey data. Actigraphic sleep parameters were extracted and analyzed the day of the Week 2 session. In Week 2, we conducted the *feedback* session. For families in the experimental arm, this session was broken down into three sections: (a) sleep environment and sleep health education, (b) sleep environment and sleep health co-evaluation, and (c) sleep environment and sleep health goal selection and behavior change strategies. The education section covered the benefits of good sleep, established recommendations for ideal child and adult sleep health through discussion, a short educational video, and informational handouts. While providing recommendations about best practices for improving sleep health and sleep environments is important, it is not novel. The innovative aspects of this intervention were the co-evaluation, goal selection, and development of behavior change strategies that integrate aspects of HBM (Knowlden et al., 2017; Hooker et al., 2018; Alyafei and Easton-Carr, 2024), SCT (Islam et al., 2023), and cognitive behavioral interventions (Blake et al., 2017) adapted for this population and the modification of sleep hygiene practices and sleep environments. Specifically, we encouraged parents and children to (a) identify discrepancies between their current sleep conditions and the recommendations for sleep health, (b) set SMART goals for aligning their sleep behaviors and environment with recommendations for sleep health (Bjerke and Renger, 2017), (c) identify possible barriers to goals and discuss possible solutions to the barriers, and (d) track the behaviors and environmental conditions they intend to change through daily diaries. Examples of goals selected by child and parents included light exercise outside in the afternoon, no caffeinated soda after 5:00 p.m., no television after dinner, not using phones after 9:00 p.m., maintaining a consistently timed bedtime routine, relaxing music and stories before bed, stretching before bed, child's adherence to a parent-set bedtime, using ear plugs, replacing television with a sound machine, eliminating light and noise from room, not co-sleeping, and using more comfortable pillows and blankets. Additionally, in hopes of addressing some of the socioeconomic barriers to sleep hygiene, we provided items based on identified needs to improve child and parent sleep environments, such as sleep masks, portable sound machines, pillows, bedding, and fans. Finally, children and parents completed the same surveys and cognitive assessments from Week 0 and received a new paper sleep diary. Participants assigned to the waitlist control arm did not receive the intervention during the *feedback* session at Week 2; at this session, families only completed the surveys and cognitive assessment and received new paper sleep diaries.

RAs summarized families' survey and actigraphic data between Weeks 2 and 4. At the Week 4 session, families in the experimental arm completed the same surveys and cognitive tests and provided *post-intervention* data. RAs administered a semi-structured interview to co-evaluate changes in sleep hygiene practices and sleep environments and assess the intervention overall. At Week 4, families in the waitlist control condition completed the same surveys and cognitive assessments. All waitlist control families elected to receive the intervention at the conclusion of the Week 4 session, which was the same as the Week 2 *feedback session* for families in the experimental arm. In addition to keeping the Fitbits and sleep items, all families were paid $100 per session, for a possible total of $300.

### Statistical analysis

Preliminary analyses were conducted to evaluate descriptive statistics by session, intervention arm, and dyad membership (parent or child) within intervention arm for sleep actigraphy, sleep diaries, and survey data.

## Results

### Descriptive statistics

#### Sleep actigraphy

Descriptive statistics for sleep actigraphy are presented in Table 3. In the waitlist control arm, children had a mean sleep onset time on 10:33 p.m. and slept for 7 h, 56 min at baseline, while parents had sleep onset of 10:55 p.m. and slept for 6 h, 17 min at baseline. In the experimental arm, children had a mean sleep onset time of 11:23 p.m. and slept for 7 h, 23 min at baseline, and a mean sleep onset time of 10:57 p.m. and slept for 7 h, 32 min post-intervention. Parents in the experimental arm had an average sleep onset at 11:50 p.m. and slept for 6 h, 8 min at baseline, and an average sleep onset at 12:03 a.m. and slept for 5 h, 54 min post-intervention. experimental arm parents also had more consistent wake times following the intervention compared to baseline.

**Table 3:** Descriptive statistics of sleep actigraphy by sleep health dimension, intervention arm (waitlist control/experimental), and dyad membership (children/parents).

Sleep health dimension	Waitlist control		Experimental		Waitlist control				Experimental			
	All		All		Children		Parents		Children		Parents	
	Weeks 0–2	Weeks 2–4	Weeks 0–2	Weeks 2–4	Weeks 0–2	Weeks 2–4	Weeks 0–2	Weeks 2–4	Weeks 0–2	Weeks 2–4	Weeks 0–2	Weeks 2–4
Sleep duration												
Sleep hours	7.11	6.86	6.71	6.72	7.94	7.79	6.28	5.93	7.39	7.54	6.13	5.89
Sleep quality												
Sleep efficiency	89.25	89.26	88.88	89.08	89.42	89.76	89.08	88.75	89.08	89.87	88.71	88.28
WASO	55.78	52.70	51.74	50.62	62.26	54.52	49.30	50.52	56.56	53.70	47.72	48.05
Sleep timing												
Sleep onset	22:44	22:29	23:38	23:30	22:33	22:06	22:55	22:52	23:23	22:57	23:50	00:03
Wake	06:49	06:20	07:12	07:01	07:25	06:46	06:12	05:53	07:41	07:19	06:49	06:43
Sleep variability												
Var. in sleep hours	0.17	0.17	0.21	0.20	0.16	0.13	0.18	0.22	0.19	0.20	0.22	0.20
Var. in sleep onset	0.05	0.05	0.04	0.05	0.04	0.04	0.05	0.06	0.05	0.05	0.04	0.05
Var. in wake	0.17	0.14	0.23	0.21	0.15	0.13	0.19	0.16	0.22	0.23	0.23	0.18

Sleep hours are exclusive of night awakenings.

#### Sleep diaries

Table 4 displays descriptive statistics from children's and parents' sleep diaries. Corroborating actigraphy data, children in the experimental arm reported earlier sleep onset and earlier wake following the intervention, while waitlist control-arm youth had later sleep onset but earlier wake over the same period. All participants (children and parents in both experimental and waitlist control arms) reported less time in bed at the end of the study compared to the beginning.

**Table 4:** Descriptive statistics of sleep diaries by intervention arm (waitlist control/experimental) and dyad membership (children/parents).

Sleep diaries	Waitlist control		Experimental		Waitlist control				Experimental			
	All		All		Children		Parents		Children		Parents	
	Weeks 0–2	Weeks 2–4	Weeks 0–2	Weeks 2–4	Weeks 0–2	Weeks 2–4	Weeks 0–2	Weeks 2–4	Weeks 0–2	Weeks 2–4	Weeks 0–2	Weeks 2–4
Sleep duration												
Time in bed	8.73	8.35	7.86	7.46	9.95	9.26	7.71	7.25	8.60	8.39	6.97	6.53
Sleep quality												
Sleep latency	29.55	29.97	29.60	27.38	25.89	19.42	32.61	45.79	31.11	28.49	27.59	26.28
Sleep timing												
Sleep onset	21:48	22:05	23:03	23:03	21:14	21:38	22:17	22:37	22:51	22:30	23:17	23:35
Wake	06:29	06:23	06:57	06:33	07:05	06:48	05:59	05:53	07:30	7:00	06:18	06:07

Time in bed represented as hours between self-reported bedtime and wake.

#### Sleep surveys

Children's and parents' self-reported sleep survey data are presented in Tables 5, 6, respectively. Describing their sleep schedules, children in the experimental arm reported their usual weeknight bedtime as 8:30 p.m. after the intervention, compared to 9:43 p.m. before the intervention; children in the waitlist control arm had no change during this period. Importantly, children in the experimental arm reported their usual weekend bedtime as 9:42 p.m. after the intervention, compared to 11:27 p.m. before the intervention—an advancement of 1 h, 45 min; children in the waitlist control arm had a delay in their usual weekend times during the same period. On a scale from 1 to 10 (with 10 being more important), children in the experimental Arm rated the importance of sleep as 7.86 before the intervention and 9.17 after; youth in the waitlist control arm had a decrease in their estimation of the importance of sleep during the same period.

**Table 5:** Descriptive statistics of child self-reported measures by sleep health dimension and intervention arm (waitlist control/experimental).

Sleep health dimension	Waitlist control			Experimental		
	Week 0	Week 2	Week 4	Week 0	Week 2	Week 4
Sleep quality						
Sleep/wake problems	43.67	40.33	39.83	41.71	35.00	38.00
Daytime sleepiness	48.17	40.33	39.67	41.14	41.86	42.20
Sleep timing						
Usual weeknight bedtime	20:40	20:00	20:02	20:51	21:43	20:30
Usual weekday wake time	05:58	06:02	05:50	06:36	06:20	05:25
Usual weekend bedtime	22:07	21:43	22:42	23:47	23:27	21:42
Usual weekend wake time	10:20	09:19	07:06	09:39	08:19	10:10
Sleep hygiene behaviors						
Physiological	4.17	4.22	5.03	4.29	4.31	4.72
Behavioral arousal	3.50	3.50	4.94	3.71	3.10	4.27
Cognitive/emotional	4.81	4.22	5.03	3.69	4.36	4.33
Sleep environment	4.40	4.80	4.90	4.06	4.31	4.20
Sleep stability	4.08	4.32	5.21	3.40	3.46	3.58
Daytime sleep	3.83	4.17	4.17	4.71	4.93	4.80
Total	4.24	4.22	4.80	4.00	4.03	4.28
Sleep environment
General sleep environment	16.83	16.33	17.00	15.71	16.67	15.20
Bedding materials	3.00	2.67	3.00	3.29	3.33	4.00
Presence of electronics	9.67	10.67	10.67	8.29	7.50	7.60
Total	29.50	29.67	30.67	27.29	27.50	26.80
Sleep beliefs						
Importance of sleep	10.00	10.00	8.17	7.71	7.86	9.17
Sleep locus of control: internal	24.33	18.67	21.33	22.86	19.67	17.00
Sleep locus of control: external	14.67	10.00	9.33	15.86	11.00	12.00

Sleep/wake problems rated using sleep/wake problems subscale of the school sleep habits survey (Wolfson et al., 2003); higher scores indicate more problems. Daytime sleepiness measured using Cleveland adolescent sleepiness questionnaire (Spilsbury et al., 2007); higher scores represent more sleepiness. Sleep hygiene behaviors measured using adolescent sleep hygiene scale (Storfer-Isser et al., 2013); higher scores represent better sleep hygiene. Sleep environment measured using children's and adolescents' sleep environment scale (Peltz et al., 2022); higher scores indicate more sleep environment problems. Importance of sleep was measured using a single item, “On a scale from 1 to 10 (1 = not important at all, 10 = extremely important) how important do you think sleep is?” Sleep locus of control adapted from health locus of control scale (Wallston et al., 1976), multidimensional health locus of control scale (Wallston et al., 1978), and sleep locus of control scale (Vincent et al., 2004); higher scores on internal subscale indicate higher self-locus of control for sleep, and higher scores on external subscale indicate lower self-locus of control for sleep.

**Table 6:** Descriptive statistics of parent self-reported measures by sleep health dimension and intervention arm (waitlist control/experimental).

Sleep health dimension	Waitlist control			Experimental		
	Week 0	Week 2	Week 4	Week 0	Week 2	Week 4
Sleep duration						
Usual sleep hours	6.00	5.92	5.67	5.14	5.71	5.42
Sleep quality						
Usual sleep latency	38.33	37.17	45.00	33.57	20.00	12.83
PSQI global score	11.33	11.33	8.83	10.29	10.71	7.50
Daytime sleepiness	9.83	14.67	15.67	9.14	16.71	16.17
Sleep timing						
Usual bedtime	21:42	22:17	22:30	23:26	23:39	23:25
Usual wake time	05:28	05:35	05:48	05:21	06:09	05:43
Sleep hygiene behaviors						
Physiological	4.53	4.90	4.73	4.64	4.80	4.80
Behavioral arousal	3.22	3.61	3.22	3.13	3.38	4.06
Cognitive/emotional	2.61	4.00	3.83	3.90	3.91	4.53
Sleep environment	4.00	4.67	4.33	4.84	5.00	5.17
Sleep stability	4.17	3.92	3.97	3.55	3.30	3.71
Daytime sleep	5.33	5.08	5.00	4.60	4.36	4.00
Total	4.06	4.52	4.40	4.41	4.40	4.65
Sleep environment
General sleep environment	13.67	14.67	14.50	14.60	16.14	13.00
Bedding materials	2.00	2.00	2.17	2.20	2.43	2.00
Presence of electronics	10.67	12.17	11.17	10.00	10.57	9.00
Total	26.33	28.83	27.83	26.80	29.14	24.00
Sleep beliefs						
Sleep locus of control: internal	24.83	23.17	23.33	22.29	23.29	23.83
Sleep locus of control: external	8.67	8.83	9.50	9.57	10.71	11.67

Usual sleep duration, latency, and bedtime, and wake time reported on Pittsburgh Sleep Quality Index (PSQI; Buysse et al., 1989) with questions adapted to ask over previous 2 weeks. For PSQI global score, higher scores represent greater sleep disturbance. Daytime sleepiness measured using the Epworth sleepiness scale (Johns, 1992); higher scores represent more sleepiness. Sleep hygiene behaviors measured using adolescent sleep hygiene scale (Storfer-Isser et al., 2013); higher scores represent better sleep hygiene. Sleep environment measured using children's and adolescents' sleep environment scale (Peltz et al., 2022); higher scores indicate more sleep environment problems. Sleep locus of control adapted from health locus of control scale (Wallston et al., 1976), multidimensional health locus of control scale (Wallston et al., 1978), and sleep locus of control scale (Vincent et al., 2004); adaptation measured extent to which parents believed their actions could influence their child's sleep, with higher scores on internal subscale indicating higher locus of control for sleep, and higher scores on external subscale indicating lower locus of control for sleep.

For parents in the experimental arm, usual sleep latency decreased from 20 min to 12.83 min after the intervention, while latency increased for parents in the waitlist control arm. Parents in the experimental arm reported an improvement in their sleep environments following the intervention (a mean sleep environment problem score of 29.14 before the intervention compared to 24.00 after); sleep environment scores for parents in the waitlist control remained relatively steady. Across sessions, parents in the experimental arm had an average increase in their sense of an external locus of control for their children's sleep, suggesting that parents felt less of an ability to influence the sleep their children received; this same scale remained more consistent across time for parents in the waitlist control group.

### Subjective feedback from families

Informal subjective feedback on the pilot study was overwhelmingly positive. One parent stated: “It was very, very helpful. I was able to get everyone on the same [sleep] schedule.” Another said the intervention was “amazing” and made her family “more aware of their sleep.” She noted that the research assistants “listened and offered solutions,” said the intervention brought her and her child closer by providing opportunities to communicate, and pointed to the “collaborative problem-solving spirit” of the approach. She also appreciated our flexibility in scheduling and found the financial incentives helpful. Another participant said, “I didn't think sleep was that important [before the program], and now I do.” Many parents reported that they would continue to use the sleep environment products and the Fitbit devices to monitor their own and their child's sleep after completing the study.

## Discussion

Children and parents in low-income settings face barriers with regard to regular sleep hygiene practices and sleep-inhibitive bedroom environments. Poor sleep health is associated with a host of chronic physical health conditions such as obesity and cardiovascular disease, mental health conditions such as depression and anxiety, and impairments in cognitive functioning (Chaput et al., 2016, 2020). Behavioral interventions are promising in this population. Findings from the pilot clinical trial of the Good Nights Sleep Program suggest that sleep can be improved when children and parents commit to making two changes to their sleep hygiene behaviors and sleep environments for 2 weeks. On average, children receiving this intervention had longer sleep and fell asleep earlier and woke earlier compared to baseline. Parents in the experimental arm had decreases in their self-reported usual sleep latency and more consistent wake times, as reflected in actigraphy, in comparison to baseline. However, due to the small sample size, these results should be interpreted with caution.

Sleep health interventions range from universal sleep education to individualized treatment of sleep disorders. Universal intervention approaches provide sleep education in a standardized format over a relatively short period of time (Cassoff et al., 2013; Gruber et al., 2016; Gaskin et al., 2024). A drawback of universal interventions is that they are not tailored to overcome individual-specific barriers in sleep hygiene practices or sleep environments; participants receiving intervention in non-problem areas are unlikely to change substantially, reducing effect sizes. At the other end of the spectrum, cognitive-behavioral interventions are individually tailored, evidence-based approaches for treating clinical sleep problems (Meltzer and Mindell, 2014; Åslund et al., 2018; Bourchtein et al., 2020). Cognitive-behavioral interventions typically last multiple sessions and involve sleep education alongside bedtime fading, stimulus control, cognitive therapy, and relaxation/mindfulness components (Blake et al., 2017). Whereas, these interventions have demonstrated effectiveness in populations with clinical sleep disorders (Schlarb et al., 2018; Meltzer et al., 2021), they are generally not applied to community populations who suffer from subclinical sleep problems rooted in their sleep hygiene and sleep environments. The monetary and time cost of multisession cognitive-behavioral interventions also limit accessibility for low-income individuals. There are a small number of hybrid sleep interventions for community populations that combine sleep education with individually tailored prescriptions for changing sleep hygiene practices that are preliminary or have shown promise (Garrison and Ward, 2019; Hiscock et al., 2019; Williamson et al., 2022; Adams et al., 2023). However, these hybrid interventions for community populations focus exclusively on children's sleep as the outcome.

Sleep interventions for low-income families can benefit from addressing the unique sleep hygiene practices and sleep environments of individuals, as well as shared parent-child level components of these constructs. Sleep health, sleep hygiene practices, and sleep environments are interdependent within parent-child dyads (Dahl and El-Sheikh, 2007; Meltzer and Montgomery-Downs, 2011; El-Sheikh and Kelly, 2017; Kouros and El-Sheikh, 2017), and existing interventions have overlooked the potential health impact of working to simultaneously improve the sleep of a parent and a child in order to build shared healthy habits (Mollborn et al., 2021). Our sleep health intervention targets needed and desired changes in sleep hygiene practices and sleep environments at both the individual and parent-child dyad levels. The intervention adapts aspects of health behavior change models by educating parents and children on the benefits of quality sleep, empowering parents and child to self-select sleep goals, identifying possible barriers and personalized solutions to goal pursuit, and self-monitoring progress (Bjerke and Renger, 2017; Knowlden et al., 2017; Hooker et al., 2018; Alyafei and Easton-Carr, 2024).

Children and adults who received the intervention in our pilot showed improvements in some actigraphic measures of sleep health, including 9 more minutes of sleep on average each night for children (while sleep hours decreased for children in the waitlist control arm over the same period), earlier sleep phase for children (26-min earlier sleep onset and 22-min earlier wake), and more consistent wake times for parents. Children also reported that weekend sleep onset times advanced by 1 h, 45 min—a huge improvement that could help to curtail social jetlag. Parents also reported that their usual sleep latency decreased by 7 min after the intervention. These findings suggests that benefits in sleep can be achieved even when individuals do not complete a full list of sleep hygiene “dos and don'ts.” Actigraphic and sleep diary results also showed that parents went to bed slightly later and woke up slightly earlier post intervention. Many parents set goals of not using technology in bed or close to bedtime but also acknowledged it was a crucial way they relaxed and connected with the world. Although it is only conjecture to offer a mechanistic explanation, it is possible that parents were delaying sleep onset to keep their relaxation time and have a technology-free bedtime routine. Such a strategy appears to have some evidence for benefit: parents had slightly truncated sleep windows, but they experienced decreased usual sleep latency. Findings with parent sleep reflect the complexities of applying health behavior change, especially for sleep health (Gregory et al., 2025; Hale and Williamson, 2026).

Children in the intervention arm slept longer, went to bed earlier, and woke up earlier post intervention compared to pre-intervention. Such improvements were not evident in the waitlist control arm. Advanced sleep windows paired with increases in sleep duration is a crucial improvement for pre-pubescent children. As children age into high school, early school start times paired with a delayed circadian rhythm interact to severely limit adolescent's sleep (Crowley et al., 2018). It appears that children who participated in our intervention were able to achieve a sleep schedule and duration that is aligned with future sleep expectations.

A portion of children in our pilot sample had multiple sleep environments due to shared parental custody or caregiving arrangements that involved family members outside the home (e.g., sleeping at a grandparent's house several nights a week), which we were not able to account for due to insufficient information about sleep location addresses. Multiple sleep environments are a reality for many children: approximately one-third of children of in the United States have shared physical custody between two caregivers (Meyer et al., 2022). This is an important consideration for several reasons. One, control of the sleep environment is a common component of sleep recommendations (Allen et al., 2016). The ability to provide a cool, dark, quiet room for sleep may not be possible for each of a child's caregivers; while some families can make sleep environments consistent across caregiver homes, it ought not be the expectation that this occurs in all cases. Therefore, it is pertinent for practitioners to consider that for many children the social ecology of sleep includes multiple sleep environments. In our intervention, we provided products such as noise machines and eye masks that could travel with children between homes.

Importantly, environmental factors affecting sleep are not limited to those inside the home. External ambient light and noise in neighborhoods also interfere with the sleep of children and adults and is an especially common environmental exposure for low-income families (Park et al., 2018; Hale et al., 2019; Crea, 2021; Nyarko and Xiao, 2024). Such environmental barriers could have dampened the effects of our intervention. Nonetheless, research has demonstrated that suitable sleep micro-environments (the space immediately surrounding an individual while they sleep, consisting of factors such as bedding and ambient temperature around the body) can be sufficient to improve sleep health even when larger environmental aspects cannot be managed (Will-Dryden et al., 2025). Sleep environment modifications in our intervention were primarily to such micro-environments, as products such as weighted blankets, fans, and space heaters sought to change the area immediately around children and parents, while larger environmental factors such as central heating, ventilation, and air conditioning units or neighborhood noise remained beyond control. Among families and sleep interventionists, attention to sleep micro-environments provides focus for changing what realistically can be changed.

Families experiencing economic hardship can be priced out of crucial elements of a healthy sleep environment such as high-quality, safe housing and affordable utilities (Adams et al., 2023; Crea, 2021; Gregory et al., 2025). The current intervention provided sleep items such as bedding, noise machines, fans, and high-quality earplugs for children and adults. Sleep items helped families achieve sleep environment goals and relieved some of the psychological pressure of being individually responsible for their sleep health in the context of economic realities (Tronto, 2013; Gregory et al., 2025).

While our pilot study provided preliminary evidence, the project to date has several key limitations. The first comes from the extremely small sample size for a randomized study, which was constrained by the relative size of the funding used to support the work; we urge readers to interpret results with caution due to this. Second, demographic characteristics (e.g., all enrolled parents being mothers) and geography (i.e., rural and suburban areas in the Southeast United States) of the sample could limit generalization to other populations. Third, our pilot study did not include follow-up over time to assess longevity of the results.

A clear next step is to learn from the pilot study and offer the Good Nights Sleep Program to a larger number of low-income children and parents in a Stage 2 randomized clinical trial to test the efficacy of the intervention (National Institute on Aging, 2025). Recruitment would be conducted through elementary schools served by a local health initiative, and inclusion/exclusion criteria would remain the same as the pilot study. To further enhance accessibility, families would have the option to participate in lab visits either on campus or at a community health center. To strengthen rigor of the approach in the extended project, the control arm would be matched on attention and educational materials throughout the sessions, allowing us to isolate causal mechanisms more rigorously. The program design would be augmented by follow-up data collection to determine longevity of intervention effects.

Study limitations were balanced by strengths of the pilot intervention. Importantly, the intervention takes into account the nested relationship between children's sleep and that of their parents, though such interdependence was not modeled statistically due to the small sample. A second important strength of the program is that it is tailored to individual and dyad needs. In the Good Nights Sleep Program, changes to sleep hygiene behaviors and environments are not prescribed or predetermined. Children and parents select changes they would like to make and are able to make in their lives. A key aim of the intervention is to empower children and parents to notice and decide what behaviors they are willing to change. Finally, children and parents who received the intervention in the pilot provided qualitative feedback that supports the program's effectiveness. Namely, they indicated that participation in the program increased their estimation of sleep's importance and said it helped to align sleep schedules within families. They further indicated that they appreciated the collaborative nature of the intervention, which we view as a hallmark of the program design. Accordingly, the pilot had an 87% retention rate, which is above the mean for dyadic behavioral interventions (Trivedi et al., 2013).

We hope that behavioral sleep medicine practitioners, other medical providers, and clinicians serving children and parents will take away several key points from this study. First, change in sleep health can stem from relatively brief discussions about children's and parents' sleep. Providers and clinicians can initiate conversations with families that follow the same basic formula of the intervention: providing sleep education, highlighting differences between families' current sleep practices and recommended behaviors, prompting children and parents to select a small number of sleep goals to simultaneously achieve each day over several weeks, and addressing potential barriers. Such a discussion could be initiated by a nurse, pediatrician, family therapist, social worker, or other medical or direct-service providers. Second, children and parents are motivated to make sleep behavior changes; most parents readily identified what they hoped to modify in their own sleep and in their children's sleep. We encourage interventionists to focus on small, incremental changes that are realistic and attainable by busy, working parents and their children. We likewise suggest that, as applicable, sleep health programs account for the non-independent, nested nature of sleep within families. While this program focuses only on changes to sleep hygiene behaviors and sleep environments, future parent-child dyad sleep interventions could capitalize on relational changes that might strengthen sleep health. For example, secure child-parent attachment in middle-childhood is associated with higher subjective sleep quality and sleep quality mediates the relationship between attachment and well-being for youth in middle childhood (Perpétuo et al., 2023). Additionally, the relational nature of sleep does not stop at the front door of the home. Adults who report living in neighborhoods with higher levels social cohesion have longer actigraphic sleep duration and more-optimal sleep schedules (Johnson et al., 2017), while youth who report greater neighborhood social cohesion have higher objective sleep quality (Bagley et al., 2018). Interventions and policies that strengthen neighborhood cohesion could improve sleep across households.

The Good Nights Sleep Program targets needed and desired changes in sleep hygiene practices and sleep environments at both individual and parent-child dyad levels. The program's conceptualization is based on widely used health behavior change models, and its design adapts evidence-based sleep health improvement strategies to the needs of low-income children and parents their children. Pilot data from the intervention is encouraging but requires additional testing with a larger sample.

## Data Availability

The raw data supporting the conclusions of this article will be made available by the authors, without undue reservation.

## Footnotes

1 Parents of all gender identities were invited to participate; all who enrolled happened to be female identifying.

2 Measures and procedures described here are a subset of the full investigation. They were selected to present descriptive findings of the pilot study.
